# Validation of Rapid and Economic Colorimetric Nanoparticle Assay for SARS-CoV-2 RNA Detection in Saliva and Nasopharyngeal Swabs

**DOI:** 10.3390/bios13020275

**Published:** 2023-02-15

**Authors:** María Armesto, Mathias Charconnet, José M. Marimón, Cristina Lía Fernández Regueiro, Jia Jia, Tingdong Yan, Ane Sorarrain, Marek Grzelczak, María Sanromán, Mónica Vicente, Boris Klempa, Javier Zubiria, Yuan Peng, Lei Zhang, Jianhua Zhang, Charles H. Lawrie

**Affiliations:** 1 Molecular Oncology Group, Biodonostia Research Institute, 20014 San Sebastián, Spain; 2 Sino-Swiss Institute of Advanced Technology (SSIAT), Shanghai University, Shanghai 201907, China; 3 Respiratory Infection and Antimicrobial Resistance Group, Biodonostia Research Institute, 20014 San Sebastián, Spain; 4 School of Life Sciences, Shanghai University, Shanghai 201907, China; 5 Colloidal Systems Chemistry, Centro de Física de Materiales (CSIC-UPV/EHU), 20018 San Sebastián, Spain; 6 Donostia Institute of Physics Centre (DIPC), 20018 San Sebastián, Spain; 7 Biomedical Research Center, Institute of Virology, Slovak Academy of Sciences, Dúbravská Cesta 9, Bratislava 845 05, Slovakia; 8 School of Microelectronics, Shanghai University, Shanghai 201907, China; 9 IKERBASQUE, Basque Foundation for Science, 48009 Bilbao, Spain; 10 Radcliffe Department of Medicine, University of Oxford, Oxford OX3 9DU, UK

**Keywords:** SARS-CoV-2, nanoparticle, POC diagnostic, colorimetric

## Abstract

Even with the widespread uptake of vaccines, the SARS-CoV-2-induced COVID-19 pandemic continues to overwhelm many healthcare systems worldwide. Consequently, massive scale molecular diagnostic testing remains a key strategy to control the ongoing pandemic, and the need for instrument-free, economic and easy-to-use molecular diagnostic alternatives to PCR remains a goal of many healthcare providers, including WHO. We developed a test (Repvit) based on gold nanoparticles that can detect SARS-CoV-2 RNA directly from nasopharyngeal swab or saliva samples with a limit of detection (LOD) of 2.1 × 10^5^ copies mL^−1^ by the naked eye (or 8 × 10^4^ copies mL^−1^ by spectrophotometer) in less than 20 min, without the need for any instrumentation, and with a manufacturing price of <$1. We tested this technology on 1143 clinical samples from RNA extracted from nasopharyngeal swabs (*n* = 188), directly from saliva samples (*n* = 635; assayed by spectrophotometer) and nasopharyngeal swabs (*n* = 320) from multiple centers and obtained sensitivity values of 92.86%, 93.75% and 94.57% and specificities of 93.22%, 97.96% and 94.76%, respectively. To our knowledge, this is the first description of a colloidal nanoparticle assay that allows for rapid nucleic acid detection at clinically relevant sensitivity without the need for external instrumentation that could be used in resource-limited settings or for self-testing.

## 1. Introduction

Since its identification in China in late 2019, the SARS-CoV-2-induced COVID-19 pandemic continues to overwhelm many healthcare systems and has generated a major economic burden on the world’s economies. Even with the development and widespread uptake of COVID-19 vaccination programs, most countries are still struggling to control the spread of the disease and the incidence of SARS-CoV-2 infection, and mortality continues to accrue with more than a doubling of reported cases (31 December 2021 (287,115,877 cases)–19 October 2022 (623,161,924 cases)) and more than 1 million attributed mortalities during the last year alone (https://covid19.who.int/ (accessed on 21 October 2022)). Consequently, the demand for massive scale molecular diagnostic testing of SARS-CoV-2 remains a key strategy to control the ongoing pandemic.

The global gold standard for COVID-19 clinical diagnosis remains the quantitative RT-PCR detection of SARS-CoV-2 in nasopharyngeal swabs. The vast majority of PCR testing is carried out on samples collected at remote testing centers that are transported and then processed by centralized diagnostic laboratories. Typical times from patient sampling to testing by RT-PCR are greater than 6 h, and limitations in capacity frequently occur due to the lack of suitable infrastructure and trained personnel when demand is greatest. Moreover, many low- and middle-income countries lack the necessary facilities for laboratory testing, causing a clear disparity in COVID-19 testing capabilities (average daily tests per 1000 population) of greater than 100-fold between high-income and low-income countries (https://apps.who.int/gb/COVID-19/pdf_files/2022/17_02/Item2.pdf (accessed on 21 October 2022))). Indeed, there have been >3 billion COVID-19 tests worldwide, of which only 0.4% were carried out in low-income countries, despite these countries representing 9% of the global population (source: WHO_press_release_ (28 October 2021)). Although rapid antigen tests can help fill this gap to some extent by identifying high viral titer symptomatic patients, concerns over poor performance hinder their widespread usage as a front-line diagnostic tool [1,2]. Therefore, there is a clear and immediate need to develop instrument-free, economic and easy-to-use molecular diagnostic alternatives to the PCR and antigen tests to detect the SARS-CoV-2 virus and other infectious pathogens. Reflecting this requirement, the World Health Organization (WHO) has developed the ASSURED criteria to which tests should conform (Affordable, Sensitive, Specific, User-friendly, Rapid and robust, Equipment-free and Deliverable to end-users) [3]. Moreover, they have made point-of-care (POC) molecular COVID-19 tests the highest priority category for Emergency Use Listing (EUL) (https://extranet.who.int/pqweb/vitro-diagnostics/coronavirus-disease-covid-19-pandemic-%E2%80%94-emergency-use-listing-procedure-eul-open (accessed on 21 October 2022)).

Consequently, in recent years, there has been renewed interest in alternative technologies to PCR for nucleic acid detection. For example, loop-mediated isothermal amplification (LAMP) is the most widely used alternative to PCR for SARS-CoV-2 detection [4], although poor clinical sensitivity in low viral load samples [5], the need for a separate heating device and the relatively high price point of LAMP (compared to antigen tests) have prevented the widespread uptake of this technology. Other researchers and ourselves have shown that nanoparticle-based colloidal biosensors can be used to detect nucleic acid sequences with high specificity, although, until now, this technology has suffered from poor sensitivity [6,7,8] or has required additional technologies or equipment to reach clinically relevant sensitivity [9]. Other technologies that have been developed to detect SARS-CoV-2 RNA include the use of aptamers [10] and Cas13a bound to nanoparticles [11], as well those that utilize the CRISPR/CAS system [12]. In addition to nucleic acid tests, there have been many attempts to improve protein immunoassay-based detection, including the use of magnetic beads [13,14], nanoparticle enhanced surface plasmon resonance (SPR) [15], selenium nanoparticles [16], electrochemical [17] and localized surface plasmon resonance (LSPR) nanostructures [18].

**Figure 1:**
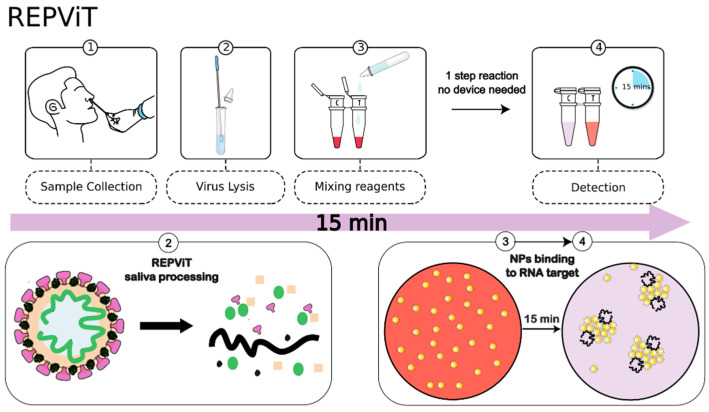
Schematic diagram of workflow of Repvit test showing change in color of solution from pink to blueish/transparent resulting from the agglomeration of nanoparticles when bound to target RNA causing plasmonic coupling, resulting in wavelength shift and optical density decrease (Figure 2) [19].

Below, we describe the development and validation of a novel colloidal nanoparticle assay technology, Repvit (*R*apid *E*conomic *P*ersonal *VI*rus *T*est), a molecular diagnostic test that can detect SARS-CoV-2 RNA directly from either nasopharyngeal swab or saliva clinical samples in less than 20 min and is detectable by the naked eye without the need for any instrumentation. This test can be used by untrained persons with a simple four-step workflow (Figure 1), needs no complicated sample treatment such as RNA extraction and can be massively scaled with a manufacturing price of <$1.

## 2. Materials and Methods

### 2.1. Test Development and Characterization

Spherical 60 nm gold nanoparticles (NanoXact in 0.05 mg/mL citrate buffer (1.5 OD)) were purchased from NanoComposix (San Diego, CA, USA) with a diameter of 61 ± 6 nm (characterized by transmission electron microscopy (JEOL 1010)), a surface charge of −60 mV and a plasmon resonance wavelength of 532 nm (characterized by dynamic light scattering (Malvern Nano ZS)). The nanoparticles were functionalized with thiolated oligonucleotides (Biomers, Ulm, Germany)) as we previously described [7,20]. In brief, nanoparticles were resuspended in 0.005% sodium dodecyl sulfate (SDS) and 0.05 M phosphate buffer pH 7.8 (PB) before adding 2 µM oligonucleotides and gradually increasing the concentration of NaCl (in SDS/PB buffer) to 0.1 M NaCl following the method of Hurst et al. [20]. After functionalization, the nanoparticles displayed an increase in hydrodynamic diameter from 69.4 nm to 73.3 nm (measured by dynamic light scattering). Oligonucleotide sequences against the E and N genes of SARS-CoV-2 were designed and selected based on thermodynamic stability, sequence homology and RNA secondary structure prediction using ViennaRNA package [21]. Candidate sequences were screened using complementary RNA target sequences, prioritized and optimized in a semi-empirical manner until selection of the final probe sequences. Final nanoparticle configurations were subsequently optimized for performance using a range of detergents and proteases with negative control saliva and nasal swab samples spiked-in with full length SARS-CoV-2 RNA. Purified full length RNA was obtained from SARS-CoV-2 Slovakia/SK-BMC5 strain prepared from Vero E6 cell culture (ECACC Catalogue No. 85020206) and supplied at a concentration of 25 ng/µL (equivalent to 1.48 × 10^9^ copies µL^−1^) and accessed through the European Virus Archive (EVAg- access code 006N-03938). This RNA was used for analytical sensitivity (limit of detection (LOD)) and analytical specificity experiments as well as a positive control during clinical validation.

For LOD determination, SARS-CoV-2-purified RNA was added to negative control saliva samples in a series of eight dilutions representing the following viral loads: 1 × 10^7^ copies mL^−1^, 3.33 × 10^6^ copies mL^−1^, 1.11 × 10^6^ copies mL^−1^, 3.75 × 10^5^ copies mL^−1^, 1.23 × 10^5^ copies mL^−1^, 4.12 × 10^4^ copies mL^−1^, 1.37 × 10^4^ copies mL^−1^, 0 copies mL^−1^ (negative). Each dilution was tested in five replicates using three separate batches of prepared nanoparticles. For each test, 20 µL of saliva was added to 17 µL of nanoparticles and 93 µL of lysis buffer (0.11% SDS, 0.11% Triton X-100, 0.58 mg/mL Proteinase K, TE buffer ). All reactions were carried out at room temperature. Absorbance measurements were performed with an Agilent BioTek Synergy 2 plate reader (Agilent, Santa Clara, CA, USA). Samples with a 540/750 nm ratio < 2.0 were considered positive, whereas those with ratio > 2.0 were considered negative. This cut-off was calculated on the basis of median values of positive and negative controls carried out in triplicate.

In addition, we carried out LOD calculations for visualization of the assay by taking photos of a series of dilutions (prepared as above), randomizing the photos and asking five independent individuals to score the photos as positive (color change) or negative (no color change). The highest dilution where the majority of observers (i.e., greater than 3/5) scored as positive was taken as the visual LOD.

For analytical specificity, in accordance with CE-regulations, we tested for potential cross-reactivity with the assay for both interfering substances and possibly confounding pathogens. For the former category we tested paracetamol solution (100 mg/mL), black Halls candies (menthol), Unnia mouth wash (Sorbitol, Phenoxyethanol, Sodium phosphate), Cinfatos (dextromethorphan hydrobromide 2 mg/mL), Disneumon nasopharyngeal spray (5 mg/mL), Dentispray (Benzocaine 50 mg/mL), Avamys nasopharyngeal spray (Fluticasone furoate), Respibien nasopharyngeal spray (oxymetazoline 0.5 mg/mL), Angileptol (chlorhexidine, benzocaine, enoxolone), Mupirocin cream (mupirocin 20 mg/g, polyethylene glycol), aspirin (500 mg pills) and Soñodor tablets (Diphenhydramide hydrochloride 50 mg pills). These compounds were homogenized (in case of solids) or added as liquid to standard positive and negative tests in triplicate. Positive tests were composed of pooled negative saliva spiked-in with 20× LOD (by spectrophotometric measurement, i.e., 1.6 × 10^6^ copies ml^−1^) and 3× LOD (2.4 × 10^5^ copies ml^−1^) SARS-COV-2-purified RNA. For cross-reactivity testing, we obtained inactivated viral or bacterial samples of the following pathogens from the Microbiology Department of Hospital University Donostia (HUD): Coronavirus OC43 strain, Coronavirus NL63 strain, Influenza A, Influenza B, Rhinovirus, Metapneumovirus, Adenovirus, Enterovirus, Respiratory Syncital virus, Legionella pneumophila, Streptococcus pneumoniae, Pseudomonas aeruginosa, Staphylococcus epidermidis, Mycopla pneumoniae, Haemophilus influenzae, Bordetella pertussis, Mycobacterium tuberculosis, Pneumocystis jirovecii, Chlamydia pneumoniae and Streptococcus pyogenes. These samples were spiked into pooled negative saliva samples and measured as described above.

### 2.2. Nasopharyngeal Sample RNA Testing

Purified RNA prepared from 188 nasopharyngeal swab samples of suspected COVID-19 patients (positive *n* = 70; negative *n* = 118) using the Allplex SARS-CoV-2 RT-qPCR assay (Seegene, Seoul, South Korea) were retrospectively obtained from the Microbiology Department of HUD. Individual patients´ details can be found in Supplementary Table S2. RNA samples were blinded to researchers before being evaluated using the Repvit assay. After 30 min, photos of the corresponding tubes were taken, and five independent observers marked the tests as positive or negative visually. The consensus opinion was recorded for each sample before unblinding the samples and comparing them to PCR results. All statistical analyses were carried out using MedCalc software (v.14.8.1).

### 2.3. Asymptomatic Saliva Testing

Saliva samples were collected from asymptomatic individuals as part of the local (Gipuzkoa, Basque Country, Spain) government screening program to identify individuals infected with SARS-CoV-2 in residential homes (*n* = 473) and in a healthcare institute (Biodonostia; *n* = 109). A further 53 saliva samples were obtained from symptomatic individuals: a total of 635 samples. Individual patients´ details are described in Supplementary Table S3. Samples were collected in universal viral transport media in dedicated containers containing a funnel with which to collect saliva. Collected samples were then transported to the Microbiology Department of HUD for PCR testing (Allplex SARS-CoV-2 assay, Seegene). Samples with a Ct-value higher than 35 were considered negative in line with local policies. Samples were collected between September 2021 and March 2022. The samples were subsequently tested in a blind fashion using the Repvit test. Twenty µL of saliva was taken from each sample and added to a tube containing 17 µL of nanoparticles and 93 µL of lysis buffer. After 20 min of incubation, the samples were spectrophotometrically measured with an Agilent BioTek Synergy 2 plate reader (Agilent, Santa Clara, CA, USA). For each 96-well plate used, the cutoff value (Abs(540 nm)/Abs(750 nm)) was calculated by ROC analysis from negative and positive control samples (negative saliva with spike-in of 1 × 10^6^ copies mL^−1^).

### 2.4. Nasopharyngeal Swab Testing

Three nasopharyngeal swab samples were prospectively collected from 320 individuals of PCR-confirmed COVID-19 status (129 positive, 191 negative). Individual patient details are given in Supplementary Table S4. One swab was used for the clinical standard test (i.e., qRT-PCR), one for antigen test and one for the Repvit test. Swabs were assigned to each test in a random manner. Testing was carried out in a blinded fashion by researchers with tubes anonymized and numerated. Tests were unblinded after deposition of the results with an independent researcher. qRT-PCR was carried out in Tongren hospital, Shanghai, according to established protocols (using the Novel Coronavirus (2019-nCoV) Nucleic Acid Detection Kit from Shanghai Biogem (Shanghai, China)). In accordance with local protocols, samples with Ct values less than 40 were considered positive. The antigen test (Novel Coronavirus (2019-nCoV) Antigen Rapid Detection kit) was obtained from Tianjing Bioscience Diagnostic Technology (Tianjing, China). For the Repvit test, nasopharyngeal swab samples were tested according to protocol and after 20 min, the color of the solution was recorded for each sample by visual inspection.

## 3. Results

### 3.1. Development and Characterization of the Test

**Figure 2:**
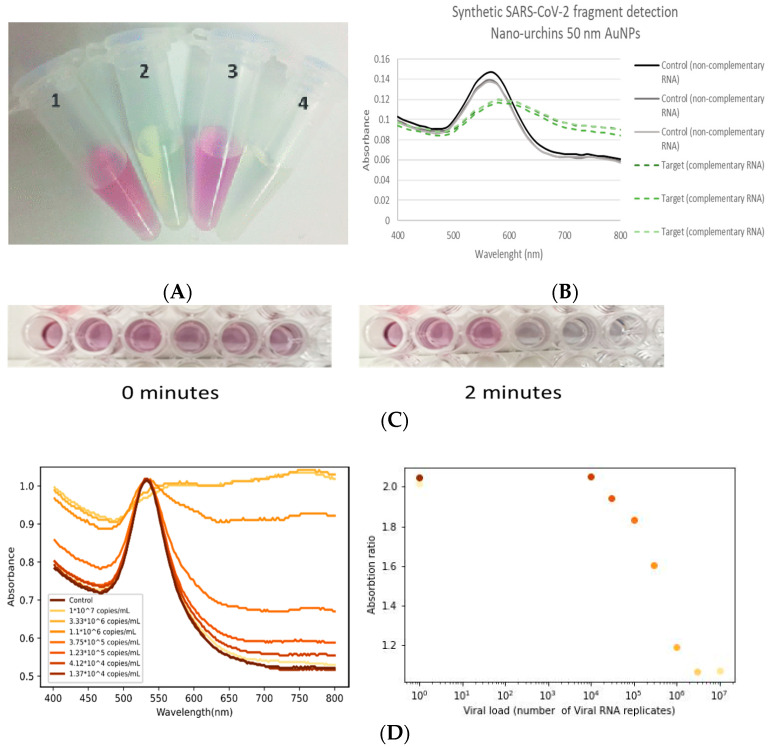
Example of detection of synthetic RNA corresponding to the complete E gene of SARS-CoV-2. (**A**) visualization of control (non-complementary RNA; tubes 1 and 3) and detection of SARS-CoV-2 E gene (5 × 10^6^ copies; tubes 2 and 4) and (**B**) corresponding spectra (repeated in triplicate) (**C**) visualization of nanoparticles in presence (three right-hand wells) of synthetic RNA corresponding to the complete N gene of SARS-CoV-2 (5 × 10^6^ copies) and control wells (three left-hand wells) containing non-complementary control RNA at time = 0 and after 2 min incubation at room temperature. (**D**) (**left**) Spectra and (**right**) corresponding values of absorption ratio (540 nm/750 nm) of detection of full length SARS-CoV-2 genomic RNA at different concentrations (30 min incubation at room temp).

We used gold nanoparticles functionalized with specific probes targeting conserved areas of the E and N genes of SARS-CoV-2 in order to develop the Repvit test [22]. As can be seen from Figure 2A,B, when the functionalized gold nanoparticles were dispersed, a red color corresponded to an absorption peak of ~540 nm. Once the nanoparticles bound to the RNA, they formed agglomerates which caused a red-shift and broadening of the absorbance spectra due to plasmonic coupling, thereby changing the solution to a transparent/blueish color (Figure 2A,B) [23]. Probe sequences were selected on the basis of a combination of thermodynamic criteria, modelled secondary structure of the RNA sequence and homology searching. Using the selected probes, we were able to detect a visible color change in 2 min in the presence of synthetic RNA fragments corresponding to the E and N genes of SARS-CoV-2 (Figure 2C). We further went on to develop a buffer system containing detergents, salts and proteinases that not only acted to stabilize the functionalized nanoparticles, but also degraded proteins in the saliva or nasopharyngeal samples matrices to release (and inactivate) the viral RNA, allowing for direct detection of SARS-CoV-2 RNA in a single solution without the need for a separate purification step. The test was further refined and optimized to detect whole length genomic SARS-CoV-2 RNA in COVID-19-infected clinical samples. Optimization was first carried out using pooled clinical samples and then individual samples, before arriving at the final formulation for the assay that was used for subsequent assay characterization and clinical validation (Figure 2D).

Using this final formulation, we measured the analytical sensitivity (limit of detection (LOD)) with three different batches of the assay using a dilution series of SARS-CoV-2 RNA spiked into negative saliva and carried out in five replicate experiments. Based on a cut-off of 95% reproducibility between tests, we determined the LOD to be 8 × 10^4^ copies/mL, although it should be noted that we could detect down to 1 × 10^4^ copies in up to 40% of the replicates. In addition, we carried out an LOD assay to determine the lowest level of virus that was detectable by the naked eye, which was calculated to be 2.1 × 10^5^ copies/mL.

For analytical specificity, we tested both potentially interfering substances and related pathogens (bacterial and viral). A full list of the compounds tested can be found in materials and methods section. We found no evidence of cross-reactivity or interference with the Repvit test (Supplementary Figures S1 and S2).

### 3.2. Detection of Extracted RNA from Nasopharyngeal Swabs

**Figure 3:**
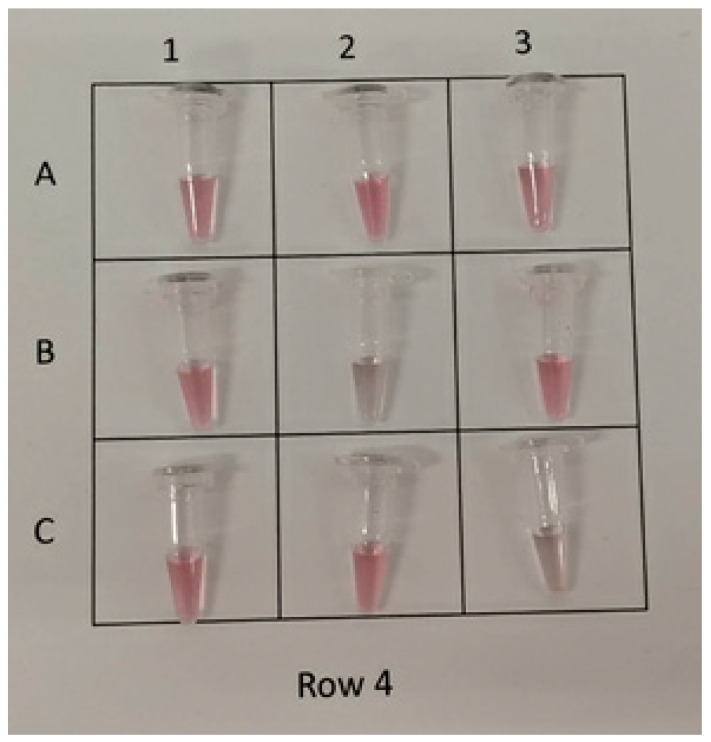
Example of nasopharyngeal RNA clinical samples used to visually score detection of presence of SARS-CoV-2 RNA. Samples B2 and C3 were scored positive and the rest of the samples negative in this example.

Purified RNA from 188 nasopharyngeal swab samples (70 positive and 118 negative) were retrospectively obtained from the Microbiology Department of HUD, which were collected from persons suspected of having COVID-19 infection between July and December 2020. Samples were anonymized before being added to the Repvit test. After 20 min of incubation at room temperature, the test solutions were photographed and sent to five independent observers for visual scoring as positive (solution color change) or negative (no color change). An example of the photo used for scoring is shown in Figure 3. Once the scoring data from the observers was collated, the consensus decision for each sample was recorded before unblinding the tests. In total, there were eight false positive and five false negative cases compared to qRT-PCR results (Supplementary Tables S1 and S2). This corresponds to a sensitivity of 92.86% (84.11–97.64%; 95% CI), a specificity of 93.22% (87.08–97.03%; 95% CI) and an accuracy of 93.09% (88.47–96.27%; 95% CI). The average Ct value of the positive samples of used in this test was 15.8 (range 11.12–32.66).

### 3.3. Asymptomatic Saliva Testing

Saliva samples were collected from 582 asymptomatic persons as a part of a COVID-19 screening program, and a further 53 saliva samples from symptomatic patients; a total cohort of 635 saliva samples (91.7% asymptomatic, 8.3% symptomatic) with 48 samples positive (7.6%) for SARS-CoV-2 and 587 negative (92.4%) by qRT-PCR. The average Ct values of positive samples was 28.03 (range 15.63–34.86). Saliva samples were retrospectively obtained from the Microbiology Department of HUD and anonymized before adding 20 µL to 93 µL lysis buffer containing detergents and proteases to inactivate the virus and dissociate the saliva matrix before placing in the Repvit solution for 20 min. After 20 min incubation at room temperature, the absorbance was measured and samples were scored as positive if the Abs(540 nm)/Abs(750 nm) ratio was less than the cut-off value (calculated by negative and positive samples), and negative if higher than that value. In total, there were twelve false positive and three false negative cases compared to qRT-PCR results (Supplementary Tables S1 and S3). This corresponds to a sensitivity of 93.75% (82.80% to 98.69%; 95% CI), a specificity of 97.96% (96.46% to 98.94%; 95% CI) and an accuracy of 97.64% (96.13% to 98.67%; 95% CI). Saliva samples from the 53 symptomatic patients were also visualized by eye and found to be concordant with the spectrophotometric results.

### 3.4. Nasopharyngeal Swab Testing

Nasopharyngeal swab samples were prospectively collected from 320 individuals with confirmed COVID-19 status (129 positive, 191 negative) from Shanghai Tongren hospital, China. Further sample swabs (×3) were taken from these individuals and individual swabs were tested by RT-PCR, an antigen test and a Repvit assay simultaneously. Both the antigen test and Repvit assay samples were scored visually in a blinded fashion before unblinding and comparison of the results with qRT-PCR. The average Ct value of the newly obtained positive samples tested by qRT-PCR was 34.35 (range 17.68–39.99). For the antigen test, there were no false positive and 112 false negative cases (and two invalid tests) compared to qRT-PCR (Supplementary Tables S1 and S4). This corresponds to a sensitivity of 13.18% (7.87–20.26%; 95% CI), a specificity of 100% (98.07–100%; 95% CI) and an accuracy of 64.78% (59.25–70.03%; 95% CI). For the Repvit assay, there were ten false positive and seven false negative cases compared to qRT-PCR (Supplementary Tables S1 and S4) corresponding to a sensitivity of 94.57% (89.14–97.79%; 95% CI), a specificity of 94.76% (90.58–97.46%; 95% CI) and an accuracy of 94.69% (91.63–96.88%; 95% CI).

## 4. Discussion

In this work, we describe the development and validation of an economic, rapid and easy-to-use molecular test to diagnose COVID-19 infection by non-trained persons that could be used with either saliva or nasopharyngeal swabs. Due to the easy-to-use workflow (Figure 1), which is similar in concept to existing antigen tests, this could easily be adapted to self-test usage, and as it requires no specialized equipment or cold-chain transport or storage conditions, the Repvit test lends itself perfectly to resource-limited situations. Moreover, as it is a chemical test based on industrially available reagents without the need for biotechnologically produced materials required for other molecular tests (i.e., enzymes in LAMP and PCR tests and antibodies in antigen tests), it is rapidly and highly scalable within existing industrial infrastructure. Furthermore, unlike antigen immunoassay tests that are reliant on the development and scaling of novel antibodies, as a nucleic acid test, the Repvit technology is rapidly adaptable to novel emerging infectious disease outbreaks or strain adaptations that can render existing antibody-based technologies inoperable. However, it should be pointed out that a recent study found that the major SARS-CoV-2 variants, to date, were detected effectively by current antigen tests based on N protein binding [24]. As the Repvit technology is based on probes that target multiple regions of the N and E genes of SARS-CoV-2, there is a lower likelihood of newly acquired mutations affecting the diagnostic ability of this assay compared with PCR or similar based technologies that rely on two primer sequences per amplicon. Indeed, we did not see any difference in the detection characteristics between the Wuhan-Hu-1, Alpha (lineage B.1.1.7), Gamma (lineage B.1.1.28.1 (P.1)) and Omicron (B.1.1.529) variants of SAR-CoV-2.

We validated the Repvit technology in a multi-center setting with both nasal swabs and saliva samples and demonstrated a performance better than that reported for antigen tests (Table 1), particularly with regard to asymptomatic samples [2].

The performance of the test between the different validation trials was similar with sensitivities of 92.86%, 93.75% and 94.57% and specificities of 93.22%, 97.96% and 94.76% in RNA samples extracted from nasopharyngeal swabs, saliva and nasopharyngeal swabs, respectively (Table 1), despite different average Ct values (15.8, 25.67 and 34.35, respectively). Presumably, this reflects chronological performance improvements in the development of the assay, although it cannot be ruled out that differences in either the sample matrix type or between the origins of the cohorts might account for this variability. The latter explanation might also explain differences between the apparently high LOD of 8 × 10^4^ copies mL^−1^ for the Repvit assay (equivalent to Ct ~25–33 [25]), compared to 10^2^–10^4^ copies mL^−1^ for PCR and 5 × 10^6^ copies mL^−1^ for antigen tests [26], and the ability of the our assay to detect multiple clinical samples with higher Ct values. Indeed, differences in the proteomic composition of saliva in individuals infected with COVID-19 have been documented [27]. It should also be noted that the LOD determined by spectrophotometric measurement was 2.6× higher than that obtained by naked eye visualization.

The use of nanoparticles as colloidal nucleic acid tests (NATs) have been described previously, however, until now they relied on electronic, optical or electrochemical signal amplification to achieve clinically relevant sensitivity [28]. Moitra et al. used gold nanoparticles attached to DNA probes targeting the N gene that resulted in visual color change in the presence of purified SARS-CoV-2 RNA when the mixture was further treated with RNase H at 65 °C [29]. However, the authors concluded that the LOD (0.18 ng µL^−1^) obtained was not sufficiently sensitive to detect clinical samples [30]. The same authors therefore added a LAMP amplification step to their protocol and achieved a sensitivity of 96.6% and a specificity of 100% with 61 nasopharyngeal samples, although details of these experiments are not provided in the publication [30]. Rodriguez-Díaz et al. recently described gold nanoparticles attached to molecular beacon-like cholesterol-containing hairpin structures to detect synthetic SARS-CoV-2 RNA transcripts, but due to sensitivity issues, a PCR-based amplification step was added in order to detect clinical samples [31]. Kumar et al. used specific oligos against the RdRp gene of SARS-CoV-2 mixed with pre-extracted viral RNA and salt before heating to 95 °C and then 60 °C [32]. This system was able to detect SARS-Cov-2 RNA with a sensitivity of 85% and specificity of 94% in a cohort of 136 nasopharyngeal samples. The Repvit technology offers nucleic acid detection at clinical sensitivity without the need for heating, a dedicated detection device or a separate RNA extraction step.

Multi-step RNA extraction is a bottleneck in molecular testing that has been addressed by several groups recently in order to reduce assay turnaround times and consumable usage to improve the usability of tests [33,34,35]. We developed a single buffer detection system containing a combination of proteases and detergents that not only disrupts the viral membrane allowing for the detection of SARS-CoV-2 viral RNA but also reduces the high protein content of the sample matrices (i.e., saliva and nasopharyngeal swabs), thereby preventing non-specific fouling of the nanoparticles, a well-known problem of nanoparticle detection systems [36]. The inclusion of SDS and Triton X-100 (amongst other detergents) in the buffer system acts to inactivate the SARS-CoV-2 virus, thereby reducing the biohazard risk of the test [37]. Moreover, the addition of detergents enhances protease activity and also acts to stabilize the functionalized gold nanoparticles [38].

Nevertheless, the current research has several limitations including it being a colorimetric test, which therefore gives a qualitative output relying on subjective visual interpretation that could lead to user errors, particularly when used by non-trained personnel or by persons with visual impairments. Indeed, it should be noted that the best performance for specificity, although not sensitivity, was obtained when we used spectrophotometric means to determine whether samples were positive or not (i.e., asymptomatic saliva samples). Consequently, we are currently developing a simple and economic spectrophotometric reader that could be used to remove the subjectivity of the result and thereby enhance the performance of the assay. Furthermore, although the test compares very favorably against the analytical sensitivity of most antigen tests, it is still several magnitudes away from the sensitivity of PCR. This is a limitation of the chemical amplification system used by the test compared to the enzymatic amplification of PCR and similar systems (e.g., LAMP). Nevertheless, we believe the Repvit test has a great deal of potential for mass screening and triage as well as for self-testing and its use within resource-limited settings.

In summary, we have developed a new rapid molecular diagnostic POC assay with better performance characteristics than antigen tests used in this study, that is readily adaptable to detect many infectious diseases, both current and emerging, without the infrastructure requirements or high costs of other molecular diagnostic techniques. This will allow for its global use as a tool in the ongoing battle between mankind and infectious pathogens.

## Figures and Tables

**Table 1:** Summary table of Repvit test performance with different clinical sample types.

	Nasopharyngeal RNA (n = 188)		Saliva Samples (n = 635)		Nasopharyngeal Swabs (n = 320)		Average (n = 1143)
	Value	95% CI	Value	95% CI	Value	95% CI	
Sensitivity	92.86%	84.11–97.64%	93.75%	82.80–98.69%	94.57%	89.14–97.79%	93.73%
Specificity	93.22%	87.08–97.03%	97.96%	96.46–98.94%	94.76%	90.58–97.46%	95.31%
Accuracy	93.09%	88.47–96.27%	97.64%	96.13–98.67%	94.69%	91.63–96.88%	95.14%

## Data Availability

All data associated with this study are available in Supplementary Materials.

## Supplementary Materials

The following supporting information can be downloaded at: https://www.mdpi.com/article/10.3390/bios13020275/s1.
