# Supplementary material for: Validation of Rapid and Economic Colorimetric Nanoparticle Assay for SARS-CoV-2 RNA Detection in Saliva and Nasopharyngeal Swabs
Source: Biosensors (Basel). 2023 Feb 15;13(2):275. doi: 10.3390/bios13020275 (PMC9954569; doi:10.3390/bios13020275)
Supplement: Supplementary file 1 [file biosensors-13-00275-s001.zip › biosensors-2173421-supplementary.pdf]

biosensors

Supplementary Materials

Validation of Rapid and Economic Colorimetric Nanoparticle Assay for SARS-CoV-2 RNA Detection in Saliva and Nasopharyngeal Swabs

María Armesto [1], Mathias Charconnet [2], José M. Marimón [3], Cristina Lía Fernández Regueiro[2], Jia Jia [2,4] Tingdong Yan [4], Ane Sorarrain [3], Marek Grzelczak [5,6], María Sanromán [5,6], Mónica Vicente [1], Boris Klempa [7], Javier Zubiria [2], Yuan Peng [2], Lei Zhang [2], Jianhua Zhang [2,8,*] and Charles H. Lawrie [1,2,9,10,*]

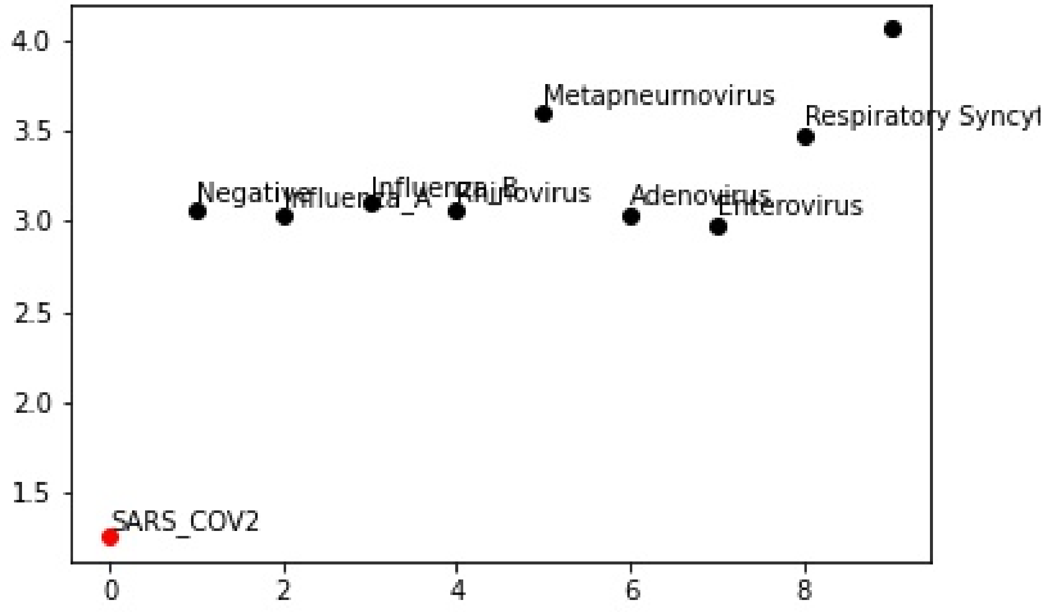

Figure S1. – Examples of analytical specificity testing with various viruses using the Repvit test with SARS-CoV-2 specific probes. Values shown on y-axis are absorbance ratios of 540nm/750nm.

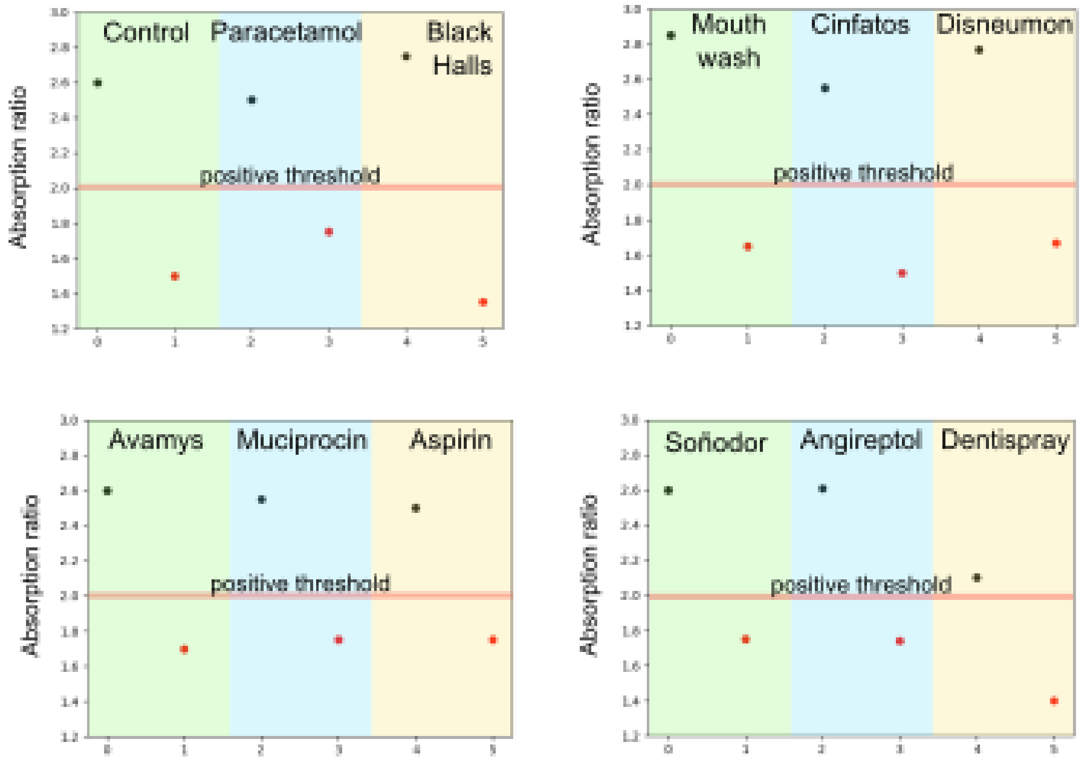

Figure S2. – Examples of analytical specificity testing with various potentially cross-contaminating substances. Values shown on y-axis are absorbance ratios of 540nm/750nm.

Table S1. Summary performance data of Repvit assay in three clinical validation sample sets in comparison to qRT-PCR. .

**qRT-PCR**	**Repvit test (nasopharyngeal RNA)**		
	**Positive**	**Negative**	**Total**
**Positive**	65	5	70
**Negative**	8	110	118
**Total**	73	115	188

**qRT-PCR**	**Repvit test (saliva samples)**		
	**Positive**	**Negative**	**Total**
**Positive**	45	3	48
**Negative**	12	575	587
**Total**	57	578	635

**qRT-PCR**	**Repvit test (nasopharyngeal swabs)**		
	**Positive**	**Negative**	**Total**
**Positive**	122	7	129
**Negative**	10	181	191
**Total**	132	188	320

Table S2. Individual sample test results of nasopharyngeal RNA by qRT-PCR and Repvit test.

Sample No.	Repvit*	PCR Ct values
1	+	16.01
2	-	Neg
3	+	16.16
4	-	Neg
5	-	Neg
**6**	**(+)**	**Neg**
7	+	15.55
**8**	**(+)**	**Neg**
9	-	Neg
10	+	Pos(NK)
11	+	11.12
12	-	Neg
13	-	Neg
14	-	Neg
15	-	Neg
16	-	Neg
17	+	15.49
18	+	13.11
19	-	Neg
20	-	Neg
21	-	Neg
22	-	Neg
23	+	16.55
24	+	Pos(NK)
25	-	Neg
26	-	Neg
**27**	**(-)**	**16.12**
28	-	Neg
29	+	14.56
**30**	**(-)**	**18.47**
31	+	16.61
32	-	Neg
33	-	Neg
34	-	Neg
35	+	Pos(NK)
36	+	16.25
37	-	Neg
38	-	Neg
39	+	11.3
40	+	15.86

41	-	Neg
42	+	16.78
43	+	15.85
44	-	Neg
45	-	Neg
46	-	Neg
47	+	Pos(NK)
48	-	Neg
49	-	Neg
**50**	**(-)**	**12.15**
51	+	12.62
52	+	16.56
53	-	Neg
54	-	Neg
55	+	17.21
56	+	Pos(NK)
57	+	Pos(NK)
58	+	Pos(NK)
59	-	Neg
60	-	Neg
61	-	Neg
62	+	32.66
63	-	Neg
64	-	Neg
65	-	Neg
66	-	Neg
67	-	Neg
68	-	Neg
69	-	Neg
70	-	Neg
71	+	Pos(NK)
72	-	Neg
73	-	Neg
74	+	Pos(NK)
75	-	Neg
76	-	Neg
77	-	Neg
78	-	Neg
79	+	15.07
80	-	Neg
81	+	16.3
82	+	16.16

**83**	**(+)**	**Neg**
84	-	Neg
85	+	Pos(NK)
**86**	**(+)**	**Neg**
87	-	Neg
88	-	Neg
**89**	**(+)**	**Neg**
90	-	Neg
91	+	Pos(NK)
92	-	Neg
93	-	Neg
94	+	14.15
95	+	13.6
96	-	Neg
97	-	Neg
98	-	Neg
99	-	Neg
100	+	13.23
101	+	Pos(NK)
**102**	**(-)**	**25.04**
103	-	Neg
104	+	16.35
105	-	Neg
106	+	Pos(NK)
107	+	12.87
108	-	Neg
109	-	Neg
110	-	Neg
111	-	Neg
112	-	Neg
113	-	Neg
114	-	Neg
115	+	16.85
116	+	Pos(NK)
117	+	Pos(NK)
118	-	Neg
119	-	Neg
120	-	Neg
121	-	Neg
122	+	12.86
123	+	12.44
124	-	Neg

125	-	Neg
126	-	Neg
127	-	Neg
128	-	Neg
129	+	Pos(NK)
130	-	Neg
131	-	Neg
132	-	Neg
133	+	15.92
**134**	**(+)**	**Neg**
135	-	Neg
136	-	Neg
137	-	Neg
138	-	Neg
139	-	Neg
140	+	14.04
141	+	13.07
142	-	Neg
**143**	**(+)**	**Neg**
144	+	Pos(NK)
145	+	12.91
146	-	Neg
147	+	Pos(NK)
148	-	Neg
149	+	Pos(NK)
150	+	16.76
151	-	Neg
152	-	Neg
153	+	16.44
154	-	Neg
155	-	Neg
156	-	Neg
157	-	Neg
158	-	Neg
159	-	Neg
160	-	Neg
161	+	15.62
162	-	Neg
163	-	Neg
164	+	Pos(NK)
165	-	Neg
166	-	Neg

167	-	Neg
168	-	Neg
169	-	Neg
170	-	Neg
171	-	Neg
172	-	Neg
173	-	Neg
174	+	Pos(NK)
175	+	16.72
176	-	Neg
177	-	Neg
178	+	16.75
179	+	13.18
180	-	Neg
181	+	23.14
**182**	**(-)**	**15.85**
183	+	12.42
184	+	14.21
185	+	Pos(NK)
186	-	Neg
187	+	Pos(NK)
**188**	**(+)**	**Neg**

*Samples evaluated by the Repvit test were scored visually by five independent observers as being either negative (no color change) or positive (color change). In case of disparity between different observers, the most frequent value was reported. Pos(NK) – Ct value unavailable for this sample. Discordant samples between PCR and Repvit test are depicted in bold, highlighted and in red font.

Table S3. Individual sample test results of saliva samples by qRT-PCR and Repvit test.

Sample no.	Ratio (540nm/750nm)	Cut-off	Repvit*	PCR Ct value
1	5.17	2.75	-	Neg
2	3.28	2.00	-	Neg
3	5.89	2.75	-	Neg
4	4.13	2.00	-	Neg
5	4.76	3.33	-	Neg
6	4.06	3.33	-	Neg
7	3.57	3.33	-	Neg
8	4.67	2.38	-	Neg
9	3.94	2.00	-	Neg
**10**	**1.63**	**2.00**	(+)	**Neg**
11	3.94	2.00	-	Neg
12	3.20	2.00	-	Neg
13	3.58	3.33	-	Neg
14	3.96	2.38	-	Neg
15	3.47	2.38	-	Neg
16	3.25	2.38	-	Neg
17	3.57	2.00	-	Neg
18	2.83	2.75	-	Neg
19	4.09	3.33	-	Neg
20	1.98	2.00	+	19.02
21	>10	2.00	-	Neg
22	3.79	2.38	-	Neg
23	3.84	2.71	-	Neg
24	3.19	2.00	-	Neg
25	5.56	2.75	-	Neg
26	3.49	3.33	-	Neg
27	4.44	2.71	-	Neg
28	4.53	2.97	-	Neg
**29**	**2.93**	**2.97**	(+)	**Neg**
30	3.26	2.00	-	Neg
31	4.43	2.75	-	Neg
32	3.69	2.71	-	Neg
33	3.79	2.00	-	Neg
34	2.18	2.00	-	Neg
35	3.56	2.38	-	Neg
36	>10	2.00	-	Neg
37	3.97	2.97	-	Neg
38	4.57	2.00	-	Neg
39	4.05	2.00	-	Neg
40	4.66	3.33	-	Neg

41	4.09	2.00	-	Neg
42	4.20	2.75	-	Neg
43	4.10	2.38	-	Neg
44	3.94	3.33	-	Neg
45	3.98	2.00	-	Neg
46	4.05	2.38	-	Neg
47	3.61	2.38	-	Neg
48	4.41	3.33	-	Neg
49	4.58	2.38	-	Neg
50	4.81	2.00	-	Neg
51	3.82	2.97	-	Neg
52	3.92	2.38	-	Neg
53	4.00	3.33	-	Neg
54	4.43	2.71	-	Neg
55	4.27	2.38	-	Neg
56	3.97	2.00	-	Neg
57	3.72	2.38	-	Neg
58	3.68	2.38	-	Neg
59	4.09	2.38	-	Neg
**60**	**1.62**	**2.97**	(+)	**Neg**
61	4.22	2.00	-	Neg
62	4.18	2.75	-	Neg
63	3.14	2.75	-	Neg
64	4.72	3.33	-	Neg
65	3.82	2.75	-	Neg
66	3.73	2.00	-	Neg
67	5.56	2.75	-	Neg
**68**	**4.10**	**2.71**	(-)	**24.9**
69	4.00	2.75	-	Neg
70	4.98	2.75	-	Neg
71	4.64	2.75	-	Neg
72	4.05	2.71	-	Neg
73	3.76	3.33	-	Neg
74	3.40	3.33	-	Neg
75	3.16	2.71	-	Neg
76	3.52	2.00	-	Neg
77	3.36	2.71	-	Neg
78	4.97	2.75	-	Neg
**79**	**2.63**	**2.75**	(+)	**Neg**
80	>10	2.00	-	Neg
81	3.49	2.38	-	Neg
82	4.22	2.00	-	Neg

83	4.07	2.75	-	Neg
84	3.22	2.71	-	Neg
85	3.44	2.71	-	Neg
86	3.34	2.38	-	Neg
87	3.84	2.00	-	Neg
88	3.93	2.97	-	Neg
**89**	**2.61**	**2.75**	**(+)**	**Neg**
90	1.88	2.00	+	30.55
91	3.37	2.97	-	Neg
92	4.75	2.75	-	Neg
93	4.07	2.71	-	Neg
94	3.97	3.33	-	Neg
95	4.23	2.00	-	Neg
96	4.72	2.00	-	Neg
97	3.55	2.00	-	Neg
98	4.64	2.75	-	Neg
99	3.32	2.00	-	Neg
100	3.70	2.00	-	Neg
101	5.11	2.97	-	Neg
102	3.16	2.97	-	Neg
103	3.06	2.71	-	Neg
104	3.02	2.38	-	Neg
105	1.75	2.00	+	23.16
106	>10	2.00	-	Neg
107	4.49	3.33	-	Neg
108	4.03	2.71	-	Neg
109	1.79	2.00	+	22.39
110	5.51	2.75	-	Neg
111	3.97	2.38	-	Neg
112	5.38	2.75	-	Neg
113	3.66	2.38	-	Neg
114	1.52	2.00	+	19.32
115	4.87	2.75	-	Neg
116	3.17	2.97	-	Neg
117	4.47	2.75	-	Neg
118	3.63	2.00	-	Neg
119	3.60	2.00	-	Neg
120	3.87	2.00	-	Neg
121	2.86	2.75	-	Neg
122	2.70	2.00	-	Neg
123	3.55	3.33	-	Neg
124	3.55	2.00	-	Neg

125	5.73	2.75	-	Neg
126	2.67	2.00	-	Neg
127	4.46	2.71	-	Neg
128	3.73	2.00	-	Neg
129	2.21	2.00	-	Neg
130	4.58	2.97	-	Neg
131	4.79	2.71	-	Neg
132	2.77	3.33	+	30.84
133	6.00	2.75	-	Neg
134	2.84	2.75	-	Neg
135	5.77	2.75	-	Neg
136	3.55	2.71	-	Neg
137	3.65	3.33	-	Neg
138	4.99	2.75	-	Neg
139	3.76	2.38	-	Neg
140	4.53	3.33	-	Neg
141	3.68	2.00	-	Neg
142	3.87	2.00	-	Neg
143	3.01	2.75	-	Neg
144	3.71	2.00	-	Neg
145	3.71	2.00	-	Neg
146	4.07	2.38	-	Neg
147	4.99	2.00	-	Neg
148	4.37	3.33	-	Neg
149	4.14	2.71	-	Neg
150	1.15	2.00	+	13.75
**151**	**2.58**	**2.97**	**(+)**	**Neg**
152	3.50	2.71	-	Neg
153	5.43	2.75	-	Neg
154	4.04	2.71	-	Neg
155	3.02	2.71	-	Neg
156	3.49	2.75	-	Neg
157	4.05	2.75	-	Neg
158	2.82	2.75	-	Neg
159	3.28	2.75	-	Neg
160	3.13	2.38	-	Neg
161	5.14	2.75	-	Neg
162	3.41	2.38	-	Neg
163	3.48	2.71	-	Neg
164	3.99	2.71	-	Neg
165	3.33	2.00	-	Neg
166	3.70	2.75	-	Neg

167	3.13	2.71	-	Neg
168	4.37	2.71	-	Neg
169	>10	2.00	-	Neg
170	4.38	2.75	-	Neg
171	3.70	2.71	-	Neg
172	3.34	3.33	-	Neg
173	4.58	2.75	-	Neg
174	2.97	2.00	-	Neg
175	3.81	2.71	-	Neg
176	3.40	2.97	-	Neg
177	1.08	2.00	+	23.15
178	2.62	2.00	-	Neg
179	4.26	3.33	-	Neg
180	2.73	2.00	-	Neg
181	3.66	2.75	-	Neg
182	3.32	2.75	-	Neg
183	3.64	2.71	-	Neg
184	1.91	3.33	+	28.86
185	1.31	2.38	+	31.88
186	4.29	2.00	-	Neg
187	3.59	2.00	-	Neg
188	3.09	2.97	-	Neg
189	3.74	2.00	-	Neg
190	3.34	2.71	-	Neg
191	1.54	2.00	+	24.96
192	3.69	2.00	-	Neg
193	4.30	2.38	-	Neg
194	2.63	2.00	-	Neg
195	3.07	2.75	-	Neg
196	2.74	2.38	-	Neg
197	5.18	2.97	-	Neg
198	5.07	2.75	-	Neg
199	5.08	3.33	-	Neg
200	3.34	2.75	-	Neg
201	3.83	2.00	-	Neg
202	4.57	2.00	-	Neg
203	3.79	2.75	-	Neg
204	4.10	2.75	-	Neg
205	3.64	2.75	-	Neg
206	2.97	2.38	-	Neg
207	3.53	2.38	-	Neg
208	4.49	2.00	-	Neg

209	2.88	2.00	-	Neg
210	4.20	2.38	-	Neg
211	5.95	2.75	-	Neg
212	4.53	3.33	-	Neg
213	3.90	2.00	-	Neg
214	1.85	2.00	+	23.39
215	1.51	2.97	+	27.15
216	4.11	2.38	-	Neg
217	5.23	2.75	-	Neg
218	4.41	2.38	-	Neg
219	3.87	2.71	-	Neg
220	5.53	2.75	-	Neg
221	5.08	2.75	-	Neg
222	3.10	2.00	-	Neg
223	2.85	2.75	-	Neg
224	3.95	2.00	-	Neg
225	5.95	2.75	-	Neg
226	4.67	3.33	-	Neg
227	5.31	2.75	-	Neg
228	2.83	2.75	-	Neg
229	4.26	2.00	-	Neg
230	4.35	2.00	-	Neg
231	4.55	2.00	-	Neg
232	3.96	2.75	-	Neg
233	3.50	2.75	-	Neg
234	2.89	2.00	-	Neg
235	4.53	2.00	-	Neg
236	4.79	3.33	-	Neg
237	3.31	2.00	-	Neg
238	1.80	2.00	+	25.86
239	3.53	2.00	-	Neg
240	3.65	2.75	-	Neg
241	3.67	2.00	-	Neg
242	5.93	2.75	-	Neg
243	2.90	2.38	-	Neg
244	3.64	3.33	-	Neg
245	2.43	2.71	+	26.86
246	4.04	2.00	-	Neg
247	3.96	2.38	-	Neg
248	1.80	2.00	+	18.63
249	4.07	2.38	-	Neg
250	4.15	2.38	-	Neg

251	3.46	2.38	-	Neg
252	3.44	2.38	-	Neg
253	5.58	2.75	-	Neg
254	4.04	2.00	-	Neg
255	3.73	2.38	-	Neg
256	3.55	2.00	-	Neg
257	4.61	2.00	-	Neg
258	2.87	2.00	-	Neg
**259**	**2.11**	**2.97**	**(+)**	**Neg**
260	3.54	2.97	-	Neg
261	4.49	3.33	-	Neg
262	3.52	2.75	-	Neg
263	4.39	2.00	-	Neg
264	2.46	2.38	-	Neg
265	4.02	3.33	-	Neg
266	4.38	2.75	-	Neg
267	3.84	3.33	-	Neg
268	3.94	2.00	-	Neg
269	4.20	2.71	-	Neg
270	3.54	2.38	-	Neg
271	3.58	2.00	-	Neg
272	3.73	2.38	-	Neg
273	3.33	3.33	+	28.24
274	3.34	2.38	-	Neg
275	1.40	2.00	+	31.39
276	3.50	2.71	-	Neg
277	4.87	2.75	-	Neg
278	3.67	3.33	-	Neg
279	3.24	3.33	+	32.93
280	2.91	2.71	-	Neg
281	4.27	2.75	-	Neg
282	3.63	2.00	-	Neg
283	4.17	2.71	-	Neg
284	3.39	2.71	-	Neg
285	4.99	2.75	-	Neg
286	3.08	2.00	-	Neg
287	>10	2.00	-	Neg
288	3.12	2.97	-	Neg
289	3.85	2.00	-	Neg
290	3.91	3.33	-	Neg
291	2.99	2.38	-	Neg
292	4.64	2.00	-	Neg

293	4.25	2.38	-	Neg
294	4.59	2.75	-	Neg
295	5.53	2.75	-	Neg
296	4.15	2.75	-	Neg
297	>10	2.00	-	Neg
298	3.74	2.71	-	Neg
299	3.10	2.00	-	Neg
300	3.78	2.38	-	Neg
301	4.67	2.00	-	Neg
302	3.50	2.00	-	Neg
303	3.41	2.00	-	Neg
304	4.63	2.97	-	Neg
305	3.95	2.00	-	Neg
**306**	**1.53**	**2.00**	(+)	**Neg**
307	5.96	2.75	-	Neg
308	4.87	3.33	-	Neg
309	4.10	2.00	-	Neg
310	5.19	2.75	-	Neg
**311**	**4.52**	**2.75**	(-)	**34.86**
312	4.42	2.71	-	Neg
313	5.13	2.75	-	Neg
314	3.67	2.00	-	Neg
315	3.92	3.33	-	Neg
316	3.97	2.38	-	Neg
317	3.52	2.00	-	Neg
318	1.44	2.00	+	18.52
319	5.34	2.75	-	Neg
**320**	**2.95**	**2.97**	(+)	**Neg**
321	5.91	2.75	-	Neg
322	2.57	2.00	-	Neg
323	3.54	2.00	-	Neg
324	>10	2.00	-	Neg
325	5.22	2.00	-	Neg
326	2.01	2.97	+	25.94
327	1.81	2.00	+	18.15
328	3.04	2.38	-	Neg
329	3.23	2.75	-	Neg
330	3.88	2.97	-	Neg
331	4.46	2.75	-	Neg
332	3.07	2.00	-	Neg
333	3.56	2.38	-	Neg
334	4.19	2.00	-	Neg

335	2.89	2.00	-	Neg
336	5.72	2.75	-	Neg
337	4.54	2.00	-	Neg
338	5.07	2.00	-	Neg
339	3.47	2.75	-	Neg
340	2.96	2.38	-	Neg
341	5.25	2.38	-	Neg
342	3.71	2.71	-	Neg
343	5.53	2.75	-	Neg
344	4.49	2.00	-	Neg
345	2.33	2.38	+	33.64
346	4.31	2.71	-	Neg
347	3.76	3.33	-	Neg
348	>10	2.00	-	Neg
349	4.12	2.00	-	Neg
350	4.60	2.75	-	Neg
351	4.11	2.75	-	Neg
352	3.82	3.33	-	Neg
353	1.13	2.00	+	16.01
354	3.93	2.71	-	Neg
355	3.44	2.71	-	Neg
356	3.71	2.75	-	Neg
357	4.81	3.33	-	Neg
358	4.18	2.75	-	Neg
359	3.57	2.71	-	Neg
360	3.84	3.33	-	Neg
361	3.75	3.33	-	Neg
362	4.13	2.71	-	Neg
363	2.67	2.38	-	Neg
364	3.83	2.38	-	Neg
365	1.27	2.00	+	15.63
366	2.85	3.33	+	26.73
367	4.32	2.38	-	Neg
368	3.37	2.97	-	Neg
369	4.20	2.97	-	Neg
370	3.68	2.00	-	Neg
371	3.86	2.38	-	Neg
372	3.46	2.75	-	Neg
373	3.58	3.33	-	Neg
374	3.45	2.38	-	Neg
375	3.53	2.00	-	Neg
376	4.22	2.38	-	Neg

377	3.95	2.38	-	Neg
378	3.68	2.97	-	Neg
379	3.53	2.00	-	Neg
380	5.61	2.75	-	Neg
381	3.47	2.38	-	Neg
382	2.70	2.71	+	31.09
383	3.69	2.38	-	Neg
384	2.00	2.75	+	25.66
385	3.35	3.33	-	Neg
386	3.99	2.75	-	Neg
387	3.27	2.00	-	Neg
388	3.59	3.33	-	Neg
389	3.36	3.33	-	Neg
390	3.01	3.33	+	32.91
391	3.19	2.75	-	Neg
392	3.11	2.71	-	Neg
393	3.67	2.71	-	Neg
394	4.20	3.33	-	Neg
395	2.68	2.00	-	Neg
396	3.44	3.33	-	Neg
397	5.13	2.00	-	Neg
398	3.41	2.75	-	Neg
399	4.27	2.71	-	Neg
400	3.10	2.75	-	Neg
401	3.70	2.00	-	Neg
402	4.02	2.71	-	Neg
403	4.30	2.71	-	Neg
404	4.56	3.33	-	Neg
405	1.62	2.00	+	23.2
406	4.95	3.33	-	Neg
407	5.91	2.75	-	Neg
408	4.48	2.00	-	Neg
409	4.08	2.71	-	Neg
410	4.39	2.71	-	Neg
411	3.61	2.00	-	Neg
412	2.61	2.00	-	Neg
413	4.29	2.00	-	Neg
414	3.18	2.00	-	Neg
415	4.28	2.38	-	Neg
416	4.76	2.00	-	Neg
417	4.56	2.71	-	Neg
**418**	**1.53**	**2.97**	**(+)**	**Neg**

419	3.55	2.75	-	Neg
420	3.34	2.38	-	Neg
421	3.61	2.75	-	Neg
422	3.62	2.38	-	Neg
423	5.76	2.75	-	Neg
424	5.62	2.75	-	Neg
425	4.16	2.71	-	Neg
426	5.61	2.75	-	Neg
427	5.06	2.75	-	Neg
428	4.41	2.00	-	Neg
429	>10	2.00	-	Neg
**430**	**2.61**	**2.97**	**(+)**	**Neg**
431	3.52	2.00	-	Neg
432	4.19	2.71	-	Neg
433	3.03	2.38	-	Neg
434	1.33	2.00	+	27.97
435	3.10	2.71	-	Neg
436	5.09	3.33	-	Neg
437	4.35	2.71	-	Neg
438	2.90	2.75	-	Neg
439	3.76	2.00	-	Neg
440	3.60	2.75	-	Neg
441	4.24	2.75	-	Neg
442	5.54	2.75	-	Neg
443	3.09	2.71	-	Neg
444	3.36	2.00	-	Neg
445	5.08	2.75	-	Neg
446	3.64	2.71	-	Neg
447	5.15	2.75	-	Neg
448	5.74	2.75	-	Neg
449	4.30	3.33	-	Neg
450	3.94	2.71	-	Neg
451	3.74	2.00	-	Neg
452	3.40	2.00	-	Neg
453	3.79	3.33	-	Neg
454	4.12	3.33	-	Neg
455	3.48	2.00	-	Neg
456	4.28	2.71	-	Neg
**457**	**3.51**	**2.38**	**(-)**	**34.05**
458	1.28	2.00	+	23.92
459	4.78	2.71	-	Neg
460	3.49	2.00	-	Neg

461	2.79	2.75	-	Neg
462	5.66	2.75	-	Neg
463	4.15	2.75	-	Neg
464	2.63	3.33	+	28.45
465	2.68	2.75	+	30.61
466	4.50	2.00	-	Neg
467	2.93	2.75	-	Neg
468	4.66	2.75	-	Neg
469	3.88	2.00	-	Neg
470	3.83	2.75	-	Neg
471	3.78	2.75	-	Neg
472	3.14	2.00	-	Neg
473	4.23	2.75	-	Neg
474	3.57	2.00	-	Neg
475	2.89	2.75	-	Neg
476	4.34	2.00	-	Neg
477	3.91	2.00	-	Neg
478	3.57	2.00	-	Neg
479	3.89	2.00	-	Neg
480	>10	2.00	-	Neg
481	2.72	2.00	-	Neg
482	3.95	2.00	-	Neg
483	4.18	2.38	-	Neg
484	4.25	2.00	-	Neg
485	3.00	2.38	-	Neg
486	3.82	2.38	-	Neg
487	5.12	2.75	-	Neg
488	4.10	2.38	-	Neg
489	3.79	3.33	-	Neg
490	3.26	2.00	-	Neg
491	4.35	2.38	-	Neg
492	4.07	2.75	-	Neg
493	3.22	2.38	-	Neg
494	3.11	2.38	-	Neg
495	4.33	3.33	-	Neg
496	5.61	2.75	-	Neg
497	4.10	2.38	-	Neg
498	3.54	2.75	-	Neg
499	3.83	2.71	-	Neg
500	3.51	2.00	-	Neg
501	3.43	3.33	-	Neg
502	3.93	2.75	-	Neg

503	4.13	2.38	-	Neg
504	4.15	2.75	-	Neg
505	4.00	2.97	-	Neg
506	4.85	2.75	-	Neg
507	4.83	2.75	-	Neg
508	4.49	2.75	-	Neg
509	2.92	2.00	-	Neg
**510**	**1.71**	**2.00**	**(+)**	**Neg**
511	4.20	3.33	-	Neg
512	3.74	3.33	-	Neg
513	2.65	2.71	+	32.13
514	3.69	2.75	-	Neg
515	1.33	2.00	+	19.41
516	2.56	2.97	+	27.5
517	2.78	2.75	-	Neg
518	4.09	2.71	-	Neg
519	2.15	2.00	-	Neg
520	1.89	2.00	+	30.48
521	4.04	2.71	-	Neg
522	3.42	2.75	-	Neg
523	4.08	2.75	-	Neg
524	2.29	2.71	+	24.67
525	1.57	2.00	+	24.21
526	4.02	2.71	-	Neg
527	4.73	2.00	-	Neg
528	4.23	2.38	-	Neg
529	4.85	2.75	-	Neg
530	4.51	2.71	-	Neg
531	3.98	2.00	-	Neg
532	4.62	2.71	-	Neg
533	4.61	2.75	-	Neg
534	5.04	2.75	-	Neg
535	4.60	2.00	-	Neg
536	4.05	2.75	-	Neg
537	4.06	2.38	-	Neg
538	3.58	2.38	-	Neg
539	3.54	3.33	-	Neg
540	4.87	2.75	-	Neg
541	3.54	2.38	-	Neg
542	2.61	2.00	-	Neg
543	2.46	3.33	+	33.59
544	3.91	2.71	-	Neg

545	4.10	2.00	-	Neg
546	3.81	2.00	-	Neg
547	3.92	2.71	-	Neg
548	3.88	3.33	-	Neg
549	3.26	2.00	-	Neg
550	3.93	2.00	-	Neg
551	3.67	2.75	-	Neg
552	4.88	2.00	-	Neg
553	4.41	2.71	-	Neg
554	3.70	2.75	-	Neg
555	1.46	2.00	+	15.52
556	3.00	2.75	-	Neg
557	3.36	2.38	-	Neg
558	3.50	2.38	-	Neg
559	4.40	2.00	-	Neg
560	3.52	2.00	-	Neg
561	2.74	2.75	+	29.92
562	4.40	2.00	-	Neg
563	3.10	2.38	-	Neg
564	3.06	2.75	-	Neg
565	4.26	2.00	-	Neg
566	4.32	2.75	-	Neg
567	3.38	2.00	-	Neg
568	3.83	2.00	-	Neg
569	3.59	2.38	-	Neg
570	4.55	2.71	-	Neg
571	3.68	2.97	-	Neg
572	3.83	2.71	-	Neg
573	1.32	2.00	+	15.78
574	3.42	2.38	-	Neg
575	3.99	2.00	-	Neg
576	2.82	2.38	-	Neg
577	4.95	3.33	-	Neg
578	3.64	3.33	-	Neg
579	3.88	2.75	-	Neg
580	4.29	2.75	-	Neg
581	3.87	2.00	-	Neg
582	4.13	2.97	-	Neg
583	5.81	2.75	-	Neg
584	4.02	2.71	-	Neg
585	3.48	2.97	-	Neg
586	3.70	3.33	-	Neg

587	3.78	2.75	-	Neg
588	3.24	2.71	-	Neg
589	3.66	2.71	-	Neg
590	>10	2.00	-	Neg
591	3.87	3.33	-	Neg
592	3.98	2.00	-	Neg
593	>10	2.00	-	Neg
594	3.49	2.00	-	Neg
595	4.27	2.97	-	Neg
596	3.70	2.38	-	Neg
597	4.36	2.00	-	Neg
598	3.53	2.38	-	Neg
599	4.66	2.75	-	Neg
600	4.95	2.75	-	Neg
601	1.81	2.00	+	24.21
602	3.95	2.71	-	Neg
603	3.60	2.00	-	Neg
604	5.45	2.75	-	Neg
605	3.48	2.38	-	Neg
606	3.49	2.00	-	Neg
607	4.35	2.75	-	Neg
608	3.08	2.00	-	Neg
609	3.33	2.00	-	Neg
610	4.19	2.00	-	Neg
611	5.48	2.75	-	Neg
612	4.22	2.71	-	Neg
613	4.59	2.38	-	Neg
614	3.22	2.75	-	Neg
615	3.07	2.75	-	Neg
616	>10	2.00	-	Neg
617	4.90	3.33	-	Neg
618	3.74	2.00	-	Neg
619	3.86	2.38	-	Neg
620	2.87	2.38	-	Neg
621	3.06	2.75	-	Neg
622	3.65	2.00	-	Neg
623	4.01	2.00	-	Neg
624	4.87	2.00	-	Neg
625	4.01	2.00	-	Neg
626	4.35	2.00	-	Neg
627	4.13	2.71	-	Neg
628	4.15	2.38	-	Neg

629	3.78	3.33	-	Neg
630	3.51	2.38	-	Neg
631	4.03	2.00	-	Neg
632	4.03	3.33	-	Neg
633	4.42	2.00	-	Neg
634	4.22	2.75	-	Neg
635	3.35	2.75	-	Neg

*Samples evaluated by the Repvit test were marked as being either negative or positive depending on the spectrophotometric ratio (Abs(540 nm)/Abs(750nm)) being above cut-off or below cut-off value respectively . Discordant samples between PCR and Repvit test are depicted in bold, highlighted and in red font.

Supplementary Table S4. Individual sample test results of nasopharyngeal swabs by qRT-PCR, antigen test and Repvit test.

Sample No.	Gender/code	Age	Antigen test	Repvit	PCR Ct value
**1**	**5165**	**NK**	**-**	(+)	**Neg**
2	5794	NK	-	-	Neg
3	female	35	(-)	+	35.94
4	female	82	+	+	30.72
5	female	90	+	+	31.17
6	male	77	(-)	+	36.12
7	9813	NK	-	-	Neg
8	771	NK	-	-	Neg
9	female	71	(-)	+	39.73
**10**	**5845**	**NK**	**-**	(+)	**Neg**
11	female	90	(-)	+	29.77
12	758	NK	-	-	Neg
**13**	**4188**	**NK**	**-**	(+)	**Neg**
14	2321	NK	-	-	Neg
15	5869	NK	-	-	Neg
16	male	59	(-)	+	37.55
17	male	40	(-)	+	39.04
18	53	NK	-	-	Neg
19	6444	NK	-	-	Neg
20	male	74	+	+	34.5
21	4263	NK	-	-	Neg
22	6873	NK	-	-	Neg
23	2150	NK	-	-	Neg
24	8769	NK	-	-	Neg
25	female	82	(-)	+	34.91
26	female	32	(-)	+	37.5
27	5894	NK	-	-	Neg
28	9919	NK	-	-	Neg
29	9260	NK	-	-	Neg
30	female	86	(-)	+	37.67
31	8095	NK	-	-	Neg
**32**	**male**	**74**	(-)	(-)	**38.08**
33	4742	NK	-	-	Neg
34	1491	NK	-	-	Neg
35	4646	NK	-	-	Neg
36	489	NK	-	-	Neg

37	male	89	(-)	+	37.31
38	6053	NK	-	-	Neg
39	8626	NK	-	-	Neg
40	male	77	(-)	+	38.17
41	male	84	(-)	+	39.86
42	3479	NK	-	-	Neg
43	3558	NK	-	-	Neg
44	female	74	(-)	+	39.47
45	7169	NK	-	-	Neg
46	6889	NK	-	-	Neg
47	female	86	(-)	+	31.87
48	female	90	(-)	+	36.53
49	2264	NK	-	-	Neg
50	male	34	(-)	+	28.84
51	6255	NK	-	-	Neg
52	509	NK	-	-	Neg
53	4222	NK	-	-	Neg
54	6419	NK	-	-	Neg
55	female	86	(-)	+	27.32
56	8130	NK	-	-	Neg
57	6428	NK	-	-	Neg
58	8714	NK	-	-	Neg
59	810	NK	-	-	Neg
60	female	78	(-)	+	37.02
61	9808	NK	-	-	Neg
62	male	65	(-)	+	33.3
63	female	3	+	+	36.68
64	903	NK	-	-	Neg
**65**	**female**	**84**	(-)	(-)	**39.94**
66	8121	NK	-	-	Neg
67	228	NK	-	-	Neg
68	321	NK	-	-	Neg
69	6608	NK	-	-	Neg
70	3831	NK	-	-	Neg
71	8347	NK	-	-	Neg
72	1635	NK	-	-	Neg
73	2275	NK	-	-	Neg
74	female	88	(-)	+	38.25
75	2579	NK	-	-	Neg
76	1548	NK	-	-	Neg
77	female	69	(-)	+	38.68
**78**	**female**	**78**	(-)	(-)	**39.36**

**79**	**male**	**34**	(-)	(-)	**30.79**
80	7737	NK	-	-	Neg
81	9805	NK	-	-	Neg
82	female	71	+	+	39.72
83	7881	NK	-	-	Neg
84	9167	NK	-	-	Neg
85	4300	NK	-	-	Neg
86	8129	NK	-	-	Neg
87	6704	NK	-	-	Neg
88	female	62	(-)	+	39.64
89	10	NK	-	-	Neg
90	4962	NK	-	-	Neg
91	female	52	(-)	+	39.85
92	7665	NK	-	-	Neg
93	female	62	(-)	+	29.96
94	male	81	(-)	+	39.72
95	female	86	(-)	+	26.92
96	female	62	(-)	+	29.65
97	3864	NK	-	-	Neg
98	male	68	(-)	+	32.81
99	female	90	(-)	+	31.83
100	4419	NK	-	-	Neg
101	1018	NK	-	-	Neg
102	1308	NK	-	-	Neg
103	male	80	(-)	+	37.72
**104**	**2868**	**NK**	-	(+)	**Neg**
105	male	54	(-)	+	38.2
106	646	NK	-	-	Neg
107	3532	NK	-	-	Neg
**108**	**4356**	**NK**	-	(+)	**Neg**
109	male	63	+	+	19.61
110	4704	NK	-	-	Neg
111	9480	NK	-	-	Neg
112	361	NK	-	-	Neg
113	2398	NK	-	-	Neg
114	2027	NK	-	-	Neg
115	female	35	(-)	+	33.04
116	1200	NK	-	-	Neg
117	4300	NK	-	-	Neg
118	male	81	(-)	+	37.56
**119**	**female**	**91**	(-)	(-)	**38.33**
120	female	70	(-)	+	32.13

121	3654	NK	-	-	Neg
122	female	70	(-)	+	39.37
123	male	68	(-)	+	34.31
124	male	59	(-)	+	33.66
125	male	75	(-)	+	39.57
126	male	80	(-)	+	36.5
127	female	91	(-)	+	33.74
128	1755	NK	-	-	Neg
129	4177	NK	-	-	Neg
130	6728	NK	-	-	Neg
131	female	82	(-)	+	34.71
132	male	81	(-)	+	39.99
133	4699	NK	-	-	Neg
134	male	63	+	+	17.68
135	7385	NK	-	-	Neg
136	2954	NK	-	-	Neg
137	female	78	(-)	+	35.59
138	1398	NK	-	-	Neg
139	female	88	(-)	+	35.63
140	4237	NK	-	-	Neg
141	5748	NK	-	-	Neg
142	female	71	(-)	+	32.01
143	3606	NK	-	-	Neg
144	female	54	(-)	+	35.89
**145**	**6242**	**NK**	**-**	(+)	**Neg**
146	4082	NK	-	-	Neg
147	1760	NK	-	-	Neg
148	2284	NK	-	-	Neg
149	6718	NK	-	-	Neg
150	female	88	(-)	+	36.68
151	7669	NK	-	-	Neg
152	5680	NK	-	-	Neg
153	4782	NK	-	-	Neg
154	7752	NK	-	-	Neg
155	male	61	(-)	+	39.1
156	6181	NK	-	-	Neg
157	female	90	(-)	+	28.93
158	7964	NK	-	-	Neg
159	3394	NK	-	-	Neg
**160**	**3492**	**NK**	**-**	(+)	**Neg**
161	male	68	(-)	+	33.57
162	5321	NK	-	-	Neg

163	female	82	(-)	+	34.34
**164**	**4701**	**NK**	**-**	(+)	**Neg**
165	male	68	(-)	+	30.46
166	5965	NK	-	-	Neg
167	female	82	(-)	+	30.67
168	5035	NK	-	-	Neg
169	female	62	(-)	+	32.51
170	2523	NK	-	-	Neg
171	5491	NK	-	-	Neg
172	female	86	(-)	+	35.44
173	9291	NK	-	-	Neg
174	7425	NK	-	-	Neg
175	female	90	+	+	26.17
176	709	NK	-	-	Neg
177	950	NK	-	-	Neg
178	3047	NK	-	-	Neg
179	6463	NK	-	-	Neg
180	female	90	(-)	+	36.57
181	female	90	(-)	+	37
182	622	NK	-	-	Neg
183	4699	NK	-	-	Neg
184	7751	NK	-	-	Neg
185	2427	NK	-	-	Neg
186	9485	NK	-	-	Neg
187	9416	NK	-	-	Neg
188	5298	NK	-	-	Neg
**189**	**4228**	**NK**	**-**	(+)	**Neg**
190	3309	NK	-	-	Neg
191	male	68	(-)	+	32.17
192	1077	NK	-	-	Neg
193	3471	NK	-	-	Neg
194	5873	NK	-	-	Neg
195	9704	NK	-	-	Neg
196	8486	NK	-	-	Neg
197	female	77	(-)	+	37.1
198	7288	NK	-	-	Neg
199	female	91	(-)	+	32.66
200	9801	NK	-	-	Neg
201	male	61	(-)	+	32.02
202	2735	NK	-	-	Neg
203	8870	NK	-	-	Neg
204	female	89	(-)	+	30.04

205	4121	NK	-	-	Neg
206	7352	NK	-	-	Neg
207	8917	NK	-	-	Neg
208	female	76	(-)	+	34.61
209	9590	NK	-	-	Neg
210	7451	NK	-	-	Neg
211	female	90	(-)	+	38.22
212	female	84	(-)	+	38.57
213	female	89	(-)	+	31.91
214	4631	NK	-	-	Neg
215	female	88	(-)	+	38.75
216	female	71	(-)	+	32.01
217	4840	NK	-	-	Neg
218	female	86	(-)	+	29.43
219	male	83	(-)	+	31.16
220	3964	NK	-	-	Neg
221	female	35	(-)	+	37.4
222	8264	NK	-	-	Neg
223	male	78	(-)	+	33.25
224	4157	NK	-	-	Neg
225	8639	NK	-	-	Neg
226	female	86	(-)	+	34.63
227	5151	NK	-	-	Neg
228	4532	NK	-	-	Neg
229	4896	NK	-	-	Neg
230	male	77	(-)	+	33.46
231	2716	NK	-	-	Neg
232	female	54	(-)	+	38.73
233	8839	NK	-	-	Neg
234	female	86	(-)	+	37.62
235	5053	NK	-	-	Neg
236	male	78	(-)	+	35.37
237	female	86	(-)	+	38.15
238	4	NK	-	-	Neg
239	male	65	(-)	+	33.11
240	male	67	(-)	+	33.22
241	3432	NK	-	-	Neg
242	3352	NK	-	-	Neg
243	2636	NK	-	-	Neg
244	male	63	(-)	+	38.55
245	3049	NK	-	-	Neg
246	834	NK	-	-	Neg

247	female	62	+	+	26.38
248	female	90	(-)	+	31.45
249	female	76	(-)	+	32.43
250	6720	NK	-	-	Neg
251	female	76	+	+	32.82
252	female	91	(-)	+	31.9
253	5179	NK	-	-	Neg
254	2695	NK	-	-	Neg
255	female	35	(-)	+	36.43
256	1715	NK	-	-	Neg
257	female	62	(-)	+	31.25
258	female	70	(-)	+	38.74
259	4080	NK	-	-	Neg
260	female	62	+	+	26.97
261	male	31	(-)	+	17.9
262	4725	NK	-	-	Neg
263	male	61	(-)	+	37.4
264	female	76	+	+	33.36
265	1238	NK	-	-	Neg
266	9390	NK	-	-	Neg
267	5042	NK	-	-	Neg
268	9491	NK	-	-	Neg
269	male	75	(-)	+	29.11
270	female	3	(-)	+	39.71
**271**	**1652**	**NK**	-	(+)	**Neg**
272	7820	NK	-	-	Neg
273	male	77	(-)	+	36.86
274	7302	NK	-	-	Neg
275	male	67	(-)	+	37.37
276	2148	NK	-	-	Neg
277	8493	NK	invalid	-	Neg
278	9163	NK	-	-	Neg
279	2096	NK	-	-	Neg
280	6330	NK	-	-	Neg
**281**	**female**	**86**	(-)	(-)	**28.17**
282	4754	NK	-	-	Neg
283	2217	NK	-	-	Neg
284	male	84	(-)	+	39.21
285	3977	NK	-	-	Neg
286	7234	NK	-	-	Neg
287	6529	NK	-	-	Neg
288	577	NK	-	-	Neg

289	3509	NK	-	-	Neg
290	female	90	+	+	27.92
291	2479	NK	-	-	Neg
292	male	59	(-)	+	38.64
293	female	91	(-)	+	33.22
294	9075	NK	invalid	-	Neg
295	male	64	(-)	+	38.55
296	7357	NK	-	-	Neg
297	female	88	(-)	+	35.47
298	3010	NK	-	-	Neg
299	male	78	(-)	+	33.91
300	female	78	(-)	+	38.54
301	female	35	(-)	+	33.62
302	2383	NK	-	-	Neg
303	77	NK	-	-	Neg
304	4339	NK	-	-	Neg
305	female	89	(-)	+	31.65
306	477	NK	-	-	Neg
307	female	91	(-)	+	34.3
308	8530	NK	-	-	Neg
309	female	89	+	+	33.32
310	5004	NK	-	-	Neg
311	female	90	+	+	28.27
312	7606	NK	-	-	Neg
313	984	NK	-	-	Neg
314	7862	NK	-	-	Neg
315	female	86	+	+	33
316	male	61	(-)	+	34.17
**317**	**female**	**90**	**+**	**(-)**	**31.45**
318	male	54	(-)	+	36.32
319	2066	NK	-	-	Neg
320	male	61	(-)	+	36.23

*Samples evaluated by the Repvit test were scored visually as being either negative (no color change) or positive (color change). Discordant samples between PCR and Repvit test are depicted in bold, highlighted and in red font. NK; not known
